# Modeling Sarcopenia to Predict Survival for Patients With Nasopharyngeal Carcinoma Receiving Concurrent Chemoradiotherapy

**DOI:** 10.3389/fonc.2021.625534

**Published:** 2021-03-11

**Authors:** Xin Hua, Wang-Zhong Li, Xin Huang, Wen Wen, Han-Ying Huang, Zhi-Qing Long, Huan-Xin Lin, Zhong-Yu Yuan, Ling Guo

**Affiliations:** ^1^ Department of Medical Oncology, SunYat-sen University Cancer Center, State Key Laboratory of Oncology in South China, Collaborative Innovation Center for Cancer Medicine, Guangdong Key Laboratory of Nasopharyngeal Carcinoma Diagnosis and Therapy, Guangzhou, China; ^2^ Department of Nasopharyngeal Carcinoma, SunYat-sen University Cancer Center, State Key Laboratory of Oncology in South China, Collaborative Innovation Center for Cancer Medicine, Guangdong Key Laboratory of Nasopharyngeal Carcinoma Diagnosis and Therapy, Guangzhou, China; ^3^ Department of Pancreatobiliary Surgery, SunYat-sen University Cancer Center, State Key Laboratory of Oncology in South China, Collaborative Innovation Center for Cancer Medicine, Guangdong Key Laboratory of Nasopharyngeal Carcinoma Diagnosis and Therapy, Guangzhou, China; ^4^ Department of Radiotherapy, SunYat-sen University Cancer Center, State Key Laboratory of Oncology in South China, Collaborative Innovation Center for Cancer Medicine, Guangdong Key Laboratory of Nasopharyngeal Carcinoma Diagnosis and Therapy, Guangzhou, China

**Keywords:** nasopharyngeal carcinoma, concurrent chemoradiotherapy (CCRT), sarcopenia, nomogram, survival

## Abstract

**Background:**

The present study aimed to construct a prognostic nomogram including Epstein-Barr virus DNA (EBV-DNA) and sarcopenia in patients with nasopharyngeal carcinoma (NPC) receiving concurrent chemoradiotherapy (CCRT).

**Methods:**

In this retrospective analysis, we studied 1,045 patients with NPC who had been treated with CCRT between 2010 and 2014. Sarcopenia was determined using routine pre-radiotherapy computed tomography scans of the third cervical vertebrae. A new S-E grade was constructed using a receiver-operating characteristic (ROC) curve analyses determined cutoff values of sarcopenia and plasma EBV-DNA. The nomogram was developed base on the sarcopenia-EBV (S-E) grade and traditional prognostic factors. A calibration curve, time-dependent ROC, decision curve analysis, and the concordance index (C-index) determined the accuracy of prediction and discrimination of the nomogram, and were compared with TNM staging system and a traditional nomogram.

**Results:**

Patient survival was significantly different when sarcopenia (P < 0.001) or EBV-DNA (P = 0.001) were used and they continued to be independent prognostic factors for survival upon univariate (P < 0.001, P = 0.002, respectively) and multivariate (P < 0.001, P = 0.015, respectively) analyses. Predicting overall survival (OS) was more accurate using the S-E grade than using TNM staging and sarcopenia or EBV-DNA alone. Nomogram B (model with sarcopenia) or nomogram A (model without sarcopenia) were then developed based on the identified independent prognostic factors. Comparing nomogram prediction with actual observation showed good agreement among the calibration curves for probability of 1-, 3-, and 5-year OS. Predicted survival (C-index = 0.77) of nomogram B was statistically higher than that of nomogram A (0.676, P = 0.020) and TNM staging (0.604, P < 0.001). Risk group stratification could distinguish between survival curves within respective TNM stages (all stages, P < 0.001; stage III, P < 0.001; stage IV, P = 0.002).

**Conclusions:**

The sarcopenia-EBV DNA nomogram allowed more accurate prediction of prognosis for patients with NPC receiving CCRT.

## Introduction

In south China, nasopharyngeal carcinoma (NPC) is endemic, with marked heterogeneity in geographical distribution, and racial and histopathological profiles (1–3). The primary treatment is radiotherapy; however, for locoregionally advanced NPC, concurrent chemoradiotherapy is the mainstay treatment (4, 5). To predict prognosis and guide treatment, the American Joint Committee on Cancer (AJCC) TNM staging system is used as the benchmark. However, the prognosis of patients with NPC at the same stage is quite different (6). The tumor-node-metastasis (TNM) staging system only grades patients according to the size of their tumor and degree of lymph node involvement, but ignores other important prognostic factors, such clinicopathological features, treatment-related factors, and nutritional status. It is believed that consideration of these factors could significantly optimize individualized prediction of survival (7–9).

Low skeletal muscle mass, termed sarcopenia, often occurs in head and neck cancer (HNC), and is associated with decreased survival in patients with HNC receiving systematic treatment with chemotherapy and radiotherapy (10–13). Sarcopenia can be conveniently determined using computed tomography (CT) simulation scans (14), which is part of the pre-treatment evaluation of radiotherapy. Sarcopenia is believed to be a compelling prognostic factor, and can potentially improve the individualized prediction of survival in HNC (15–17). Epstein-Barr virus DNA (EBV-DNA), is used widely in clinical applications and is believed to be the best complement of the TNM staging system. In patients with NPC, EBV-DNA levels correlate with tumor burden and prognosis (18–21). It is important to note, however, that no effective method has been developed to combine plasma EBV-DNA and sarcopenia to predict prognosis and guide treatment. Therefore, a comprehensive and convenient tool that combines sarcopenia, EBV-DNA, and other risk factors might represent a useful clinical decision-making tool. Therefore, the present study aimed to develop a practical prognostic tool for patients with NPC treated with concurrent chemoradiotherapy (CCRT) by incorporating sarcopenia, EBV-DNA, and known clinicopathological variables.

## Patients and Methods

### Patients

This retrospective observation study enrolled 806 patients with NPC who underwent CCRT from January 2010 to December 2014 at Sun Yat-sen University Cancer Center (SYSUCC), Guangzhou, China. For all the patients, clinical and histopathological data were obtained. We excluded 239 patients from among the initially enrolled 1,045 patients. The **Supplementary Material** provides details of the inclusion criteria and exclusion criteria. The 8^th^ AJCC TNM staging manual was used to restage all the patients. The Research Ethics Committee of SYSUCC approved this study, and all the patients provided written informed consent before treatment.

### Data Collection and Definitions

The presence of sarcopenia was evaluated using the skeletal muscle index (SMI; the skeletal muscle area (cm^2^)/square of height (m^2^) (10). The skeletal muscle area at the third cervical vertebral (C3) level was measured according to a validated method (22, 23) using CT simulation images of RT with Monaco TPS software version 5.1 (Elekta CMS, Maryland Heights, MO, USA). A senior radiotherapy oncologist (LG) provided hand-drawn pictures of the muscle contours which took about 3 to 5 minutes for one patient, the methodology of which is detailed in the **Supplementary Materials**. In general, the sketching process is relatively simple and convenient. Real-time quantitative polymerase chain reaction was used to measure the plasma EBV-DNA levels (copies/ml) (20), according to the detailed methodology shown in the **Supplementary Materials**. Primary laboratory data were collected within 7 days of diagnosis, and the patients’ medical records provided the clinicopathological data. The patients’ body mass index (BMI) was calculated (weight (kg)/square of the height in meters (m^2^)), and patients were classified as obese (BMI ≥ 25) and non-obese (BMI < 25).

### CCRT Protocol and Follow-Up

Patients were treated in accordance with the guidelines of our institute (detailed in the **Supplementary Materials**) (24), and were followed-up at least once every three months (years 1–3) and then every six months until death after treatment. Overall survival (OS) was the primary endpoint, defined as the time from the date of diagnosis to the date of death or last follow-up.

### Statistical Analysis

Statistical analyses were conducted using SPSS version 23.0 (IBM Corp., Armonk, NY, USA) and R 3.5.2 (http://www.r-project.org). The optimal cutoff points for the SMI and EBV-DNA were determined using receiver-operating characteristic (ROC) curve analysis with survival status as an endpoint. ROC curve analysis showed that for sarcopenia, the optimal cut-off points were SMI < 22.00 cm^2^/m^2^ (men) and < 18.61 cm^2^/m^2^ (women); the EBV-DNA optimal cut-off point was 2,895 copies/ml. The risk score for each patient was calculated using nomogram B and the cutoff values were determined using X-tile (https://medicine.yale.edu/lab/rimm/research/software/) by grouping the patients into three subgroups after sorting by total score (score: < 13.21, low risk; 13.21 to 16.42, middle risk; and >16.42, high risk). The Kaplan–Meier method was used to produce the survival curves, which were compared using log-rank tests. Harrell’s concordance index (C-index) and time-dependent receiver operative characteristics (tROC) were used to measure the models’ discriminative ability to predict survival. The predictive accuracy was determined from the area under the curve (AUC) of tROC curves, which plotted sensitivity versus specificity. The Cox proportional hazards model was used to perform univariate and multivariate analysis, with multivariate analysis being performed for variables with P values < 0.20 from the univariate analysis. Nomograms were formulated on the basis of the results of the multivariate analysis. C-index, decision curve analysis (DCA), and the AUC of the tROC analysis were used to measure the nomogram’s performance. The agreement between nomogram-predicted values and ideal observation in the study cohort were compared using calibration curves. The clinical utility of the predictive nomogram was assessed by quantifying the net benefits at different threshold probabilities in a decision curve.

## Results

### Patient Characteristics

**Table 1** presents the characteristics of the 806 patients enrolled in this retrospective study. At diagnosis, the median age was 45 years (range, 18–84 years). The median SMI was 24.47 cm^2^/m^2^ (range, 10.96–57.46 cm^2^/m^2^) and the median BMI was 23.2 kg/m^2^ (range, 13.48–33.9 kg/m^2^). Next, the S-E grade was defined based on the above-mentioned cutoff points as follows: S-E grade 1: Patients with an elevated SMI (non-sarcopenia) and decreased EBV-DNA; S-E grade 2: Patients with elevated or reduced scores for either of the two factors; S-E grade 3: Patients with elevated EBV-DNA and decreased SMI (sarcopenia).

**Table 1:** Patient demographics and clinical characteristics.

Characteristic	
	Number of patients (%)
**Age**	
≥45 years	394 (48.9)
<45 years	412 (51.1)
**Sex**	
Male	602(74.7)
Female	204 (25.3)
**Histological type**	
WHO I	4 (0.5)
WHO II	9 (1.1)
WHO III	793 (98.4)
**HGB**	
<113 g/liter	23 (2.9)
113–151 g/liter	521 (64.6)
≥151 g/liter	262 (32.5)
**LDH**	
≥245 U/liter	50 (6.2)
<245 U/liter	756 (93.8)
**Hs-CRP**	
<1 g/ml	295 (36.6)
1–3 g/ml	283 (35.1)
≥3 g/ml	228 (28.3)
**T stage**	
T1	39 (4.8)
T2	156 (19.4)
T3	493 (61.2)
T4	118 (14.6)
**N stage**	
N0	77 (9.6)
N1	434 (53.8)
N2	252 (31.3)
N3	43 (5.3)
**TNM stage**	
II	114 (14.1)
III	537 (66.6)
IV	155 (19.2)
**BMI**	
≥25 kg/m^2^	208 (25.8)
<25 kg/m^2^	598 (74.2)
**EBV-DNA**	
<2895 copy/ml	505 (62.7)
≥2895 copy/ml	301 (37.3)
**Sarcopenia**	
No	609 (75.6)
Yes	197 (24.4)
**S-E grade**	
grade 1	377 (46.8)
grade 2	360 (44.7)
grade 3	69 (8.6)

WHO, World Health Organization; HGB, hemoglobin; LDH, serum lactate dehydrogenase levels; hs-CRP, high-sensitivity C-reactive protein; BMI, body mass index; EBV-DNA, Epstein-Barr virus DNA; S-E grade, sarcopenia EBV-DNA grade.

### Survival Analysis of Sarcopenia, S-E grade, and EBV-DNA

**Figure 1:**
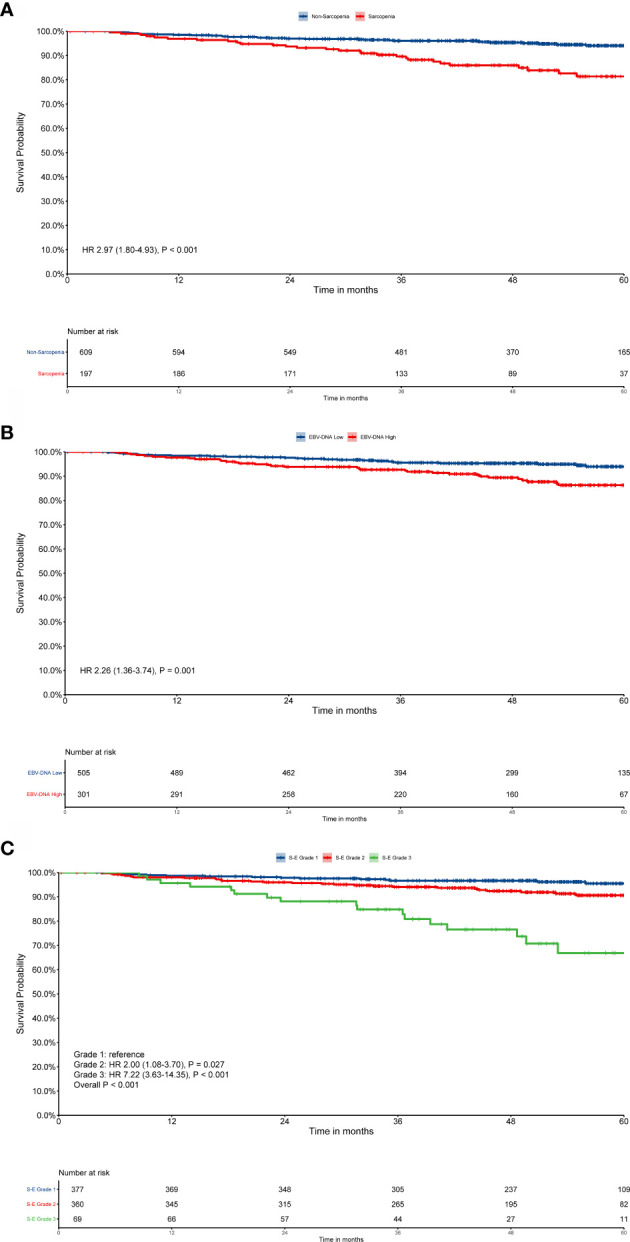
Kaplan–Meier survival curves for overall survival (OS). EBV-DNA = Epstein-Barr virus DNA; S-E grade = sarcopenia EBV-DNA grade. Kaplan–Meier curves for: **(A)** OS by sarcopenia; **(B)** OS by EBV-DNA; **(C)** OS by S-E grade. Survival curves were calculated using the Kaplan–Meier method and compared using the log-rank test.

Patient survival was significantly different when sarcopenia (P < 0.001, **Figure 1A**) or EBV-DNA (P = 0.001, **Figure 1B**) were used, as demonstrated by Kaplan–Meier analysis. The median survival time was 51.8 (interquartile range (IQR): 42.8–61.5) months for patients with grade 1 S-E, which was higher than that of patients with S-E grade 2 (49.8 months, IQR: 35.0–59.0) and S-E grade 3 (41.3 months, IQR: 31.7–53.0). For OS, patients with S-E grade 1 showed longer survival compared with patients with S-E grade 2 or S-E grade 3 (P < 0.001, **Figure 1C**). We also analyzed the prognostic relationship between S-E in relapse free survival (RFS) (P = 0.240, **Supplementary Figure S2A**) and distant metastasis free survival (DMFS) (P < 0.001, **Supplementary Figure S2B**). **Table 1** shows the univariate and multivariate analysis results for OS. T stage (P = 0.010), N stage (P = 0.003), sarcopenia (P < 0.001), and EBV-DNA (P = 0.002) were identified as prognostic factors in patients with NPC using univariate analysis. All four variables remained independent prognostic factors for survival upon multivariate analyses (P = 0.027, P = 0.005, P = 0.015, P < 0.001, respectively; **Table 2**).

**Table 2:** Univariate and multivariate analysis of overall survival.

Characteristic				
	Univariate analysis		Multivariate analysis	
	Hazard ratio (95% CI)	P	Hazard ratio (95% CI)	P
**Age**				
≥45 years	1		1	
<45 years	0.663 (0.398–1.102)	0.113	0.659 (0.394–1.104)	0.113
**Sex**				
Male	1			
Female	0.799 (0.433–1.476)	0.474		
**Histological type**				
WHO III III	1		1	
WHO I/II	3.046 (0.951–9.758)	0.061	2.960 (0.868–10.095)	0.083
**HGB**				
<113 g/liter	1			
113–151 g/liter	1.446 (0.198–10.572)	0.716		
≥151 g/liter	2.170 (0.294–15.995)	0.447		
**LDH**				
≥245 U/liter	1			
<245 U/liter	0.665 (0.266–1.661)	0.382		
**Hs-CRP**				
<1 g/ml	1		1	
1–3 g/ml	0.885 (0.463–1.691)	0.712	0.821 (0.422–1.600)	0.563
≥3 g/ml	1.587 (0.877–2.873)	0.127	1.154 (0.608–2.192)	0.661
**T stage**				
T1–2	1		1	
T3	1.628 (0.786–3.374)	0.190	1.590 (0.760–3.326)	0.218
T4	2.964 (1.296–6.779)	0.010	2.629 (1.115–6.199)	0.027
**N stage**				
N0–1	1		1	
N2	2.215 (1.304–3.763)	0.003	2.196 (1.266–3.806)	0.005
N3	3.013 (1.241–7.316)	0.015	2.255 (0.900–5.653)	0.083
**BMI**				
≥25 kg/m^2^	1			
<25 kg/m^2^	0.931 (0.532–1.630)	0.802		
**EBV-DNA**				
<2895 copy/ml	1		1	
≥2895 copy/ml	2.259 (1.362–3.746)	0.002	1.957 (1.142–3.356)	0.015
**Sarcopenia**				
No	1		1	
Yes	2.974 (1.795–4.928)	<0.001	2.974 (1.772–4.989)	<0.001

Hazard ratios estimated by Cox proportional hazards regression. All statistical tests were two-sided. CI, confidence interval; HR, hazard ratio; WHO, World Health Organization; HGB, hemoglobin; LDH, serum lactate dehydrogenase levels; hs-CRP, high-sensitivity C-reactive protein; BMI, body mass index; EBV-DNA, Epstein-Barr virus DNA.

### Prognostic Accuracy Comparisons Among TNM Stage, S-E Grade, EBV-DNA, and Sarcopenia

**Figure 2:**
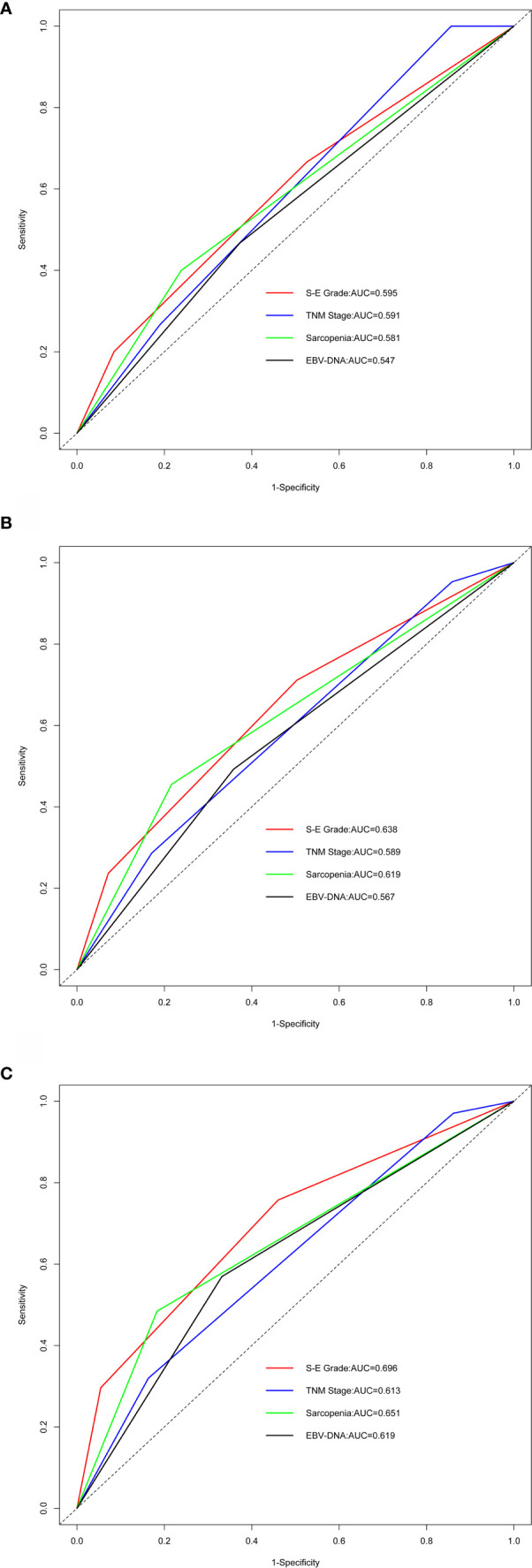
Comparing predictive ability by time-dependent receiver-operating characteristic curve (tROC) analysis. EBV-DNA = Epstein-Barr virus DNA; S-E grade = sarcopenia EBV-DNA grade; AUC = area under the receiver-operating characteristic curve. **(A–C)** Time-dependent ROC analysis for 1-, 3-, and 5-year OS, the x-axis represents the sensitivity value (true positive rate), and the y-axis represents the 1-specificity (false positive rate). The larger AUC implies that patients can obtain the maximum prediction using this model.

The C-index values for S-E grade, TNM stage, sarcopenia, and EBV-DNA were 0.663 (95% CI, 0.594–0.732), 0.604 (95% CI, 0.544–0.664), 0.623 (95% CI, 0.558–0.688), and 0.600 (95% CI, 0.535–0.665), respectively (**Supplementary Table S1**). S-E grade demonstrated significantly increased prognostic accuracy compared with the TNM stage (P < 0.001). For 1-, 3-, and 5-year survival, similar findings were obtained from the results of time-dependent ROC analysis. S-E grade had the largest AUC among the four methods (**Figure 2**), which indicated that the S-E grade was better at predicting outcomes than TNM staging and sarcopenia or EBV-DNA alone.

### Developing Nomograms With or Without S-E Grade

The majority of centers do not apply sarcopenia routinely; therefore, we constructed a traditional nomogram A (model 1) according to Tang et al. (20) and a new nomogram B (model 2) based on the S-E grade. The results of the analysis of significant prognostic factors for OS are shown in **Table 3**. For model 1, N stage, EBV-DNA and T stage were identified as independent prognostic factors using multivariate Cox-analysis. For model 2, the same analysis identified N stage, histology, T stage, and S-E grade as independent prognostic factors.

**Table 3:** Univariate and multivariate analyses of overall survival in model 1 and model 2.

Characteristic			Model 1		Model 2	
	Univariate analysis		Multivariate analysis		Multivariate analysis	
	Hazard ratio (95% CI)	P	Hazard ratio (95% CI)	P	Hazard ratio (95% CI)	P
**Age**						
≥45 years	1		1		1	
<45 years	0.663 (0.398–1.102)	0.113	0.653 (0.391–1.091)	0.104	0.633 (0.378–1.061)	0.083
**Sex**						
Male	1					
Female	0.799 (0.433–1.476)	0.474				
**Histological type**						
WHO III	1		1		1	
WHO I/II	3.046 (0.951–9.758)	0.061	3.126 (0.943–10.362)	0.062	3.453 (1.023–11.654)	0.046
**HGB**						
<113 g/liter	1					
113–151 g/liter	1.446 (0.198–10.572)	0.716				
≥151 g/liter	2.170 (0.294–15.995)	0.447				
**LDH**						
≥245 U/liter	1					
<245 U/liter	0.665 (0.266–1.661)	0.382				
**Hs–CRP**						
<1g/ml	1		1		1	
1–3 g/ml	0.885 (0.463–1.691)	0.712	0.719 (0.371–1.393)	0.328	0.800 (0.413–1.548)	0.508
≥3 g/ml	1.587 (0.877–2.873)	0.127	0.982 (0.521–1.852)	0.955	1.119 (0.598–2.094)	0.726
**T stage**						
T1–2	1		1		1	
T3	1.628 (0.786–3.374)	0.190	1.571 (0.750–3.290)	0.231	1.589 (0.759–3.327)	0.219
T4	2.964 (1.296–6.779)	0.010	3.059 (1.301–7.193)	0.010	2.594 (1.096–6.135)	0.030
**N stage**						
N0–1	1		1		1	
N2	2.215 (1.304–3.763)	0.003	2.179 (1.260–3.771)	0.005	2.144 (1.241–3.704)	0.006
N3	3.013 (1.241–7.316)	0.015	2.624 (1.062–6.485)	0.037	2.264 (0.907–5.651)	0.080
**BMI**						
≥25 kg/m^2^	1					
<25 kg/m^2^	0.931 (0.532–1.630)	0.802				
**EBV–DNA**						
<2895 copy/ml	1		1		Not selected	
≥2895 copy/ml	2.259 (1.362–3.746)	0.002	1.846 (1.079–3.159)	0.025	Not selected	
**Sarcopenia**						
No	1		Not selected		Not selected	
Yes	2.974 (1.795–4.928)	<0.001	Not selected		Not selected	
**S–E grade**						
grade 1	1		Not selected		1	
grade 2	2.000 (1.081–3.698)	0.027	Not selected		1.625 (0.868–3.044)	0.129
grade 3	7.219 (3.630–14.353)	<0.001	Not selected		5.879 (2.881–11.998)	<0.001

Hazard ratios estimated by Cox proportional hazards regression. All statistical tests were two-sided. CI, confidence interval; HR, hazard ratio; WHO, World Health Organization; HGB, hemoglobin; LDH, serum lactate dehydrogenase levels; hs-CRP, high-sensitivity C-reactive protein; BMI, body mass index; EBV-DNA, Epstein-Barr virus DNA; S-E grade, sarcopenia EBV-DNA grade.

**Figure 3:**
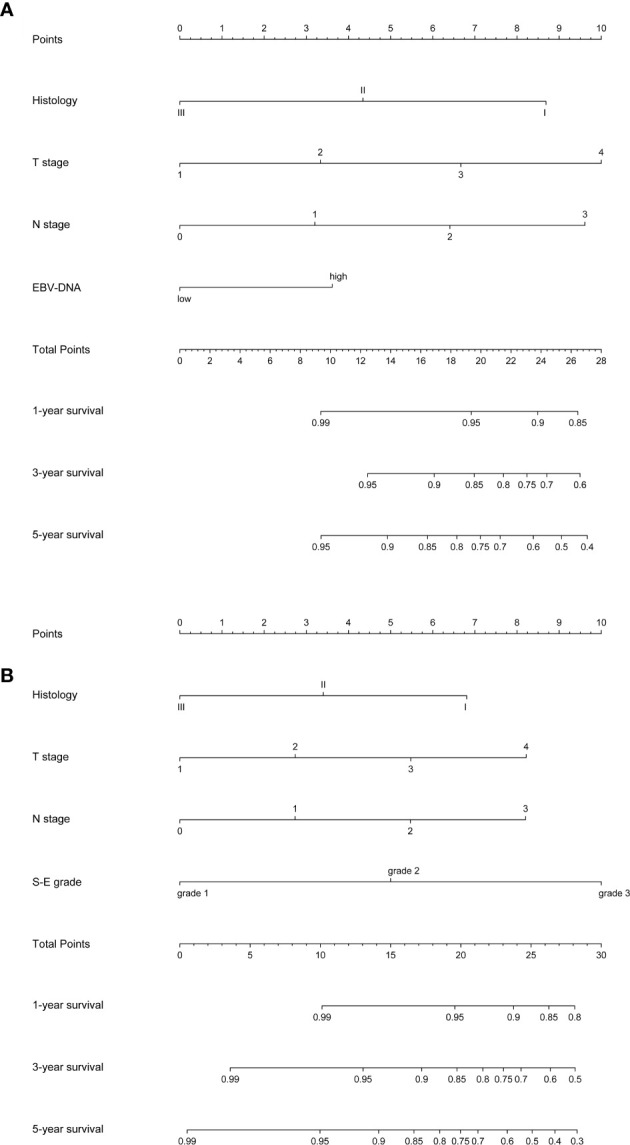
Nomograms to predict 1-, 3- and 5-year overall survival (OS). EBV-DNA = Epstein-Barr virus DNA; S-E grade = sarcopenia EBV-DNA grade. **(A)** Nomogram A, including histology, T stage, N stage, and EBV-DNA levels, for 1-, 3- and 5-year OS. The nomogram allows the user to obtain the probability of 1-, 3- and 5-year OS corresponding to a patient’s combination of covariates. As an example, locate the patient’s T stage and draw a line straight upward to the “Points” axis to determine the score associated with that T stage. Repeat the process for each variable, and sum the scores achieved for each covariate, and locate this sum on the “Total Points” axis. Draw a line straight down to determine the likelihood of 1-, 3- and 5-year OS; **(B)** Nomogram B, including histology, T stage, N stage, and S-E grade, for 1-, 3- and 5-year overall survival (OS). The nomogram allows the user to obtain the probability of 1-, 3- and 5-year OS corresponding to a patient’s combination of covariates.

**Figure 4:**
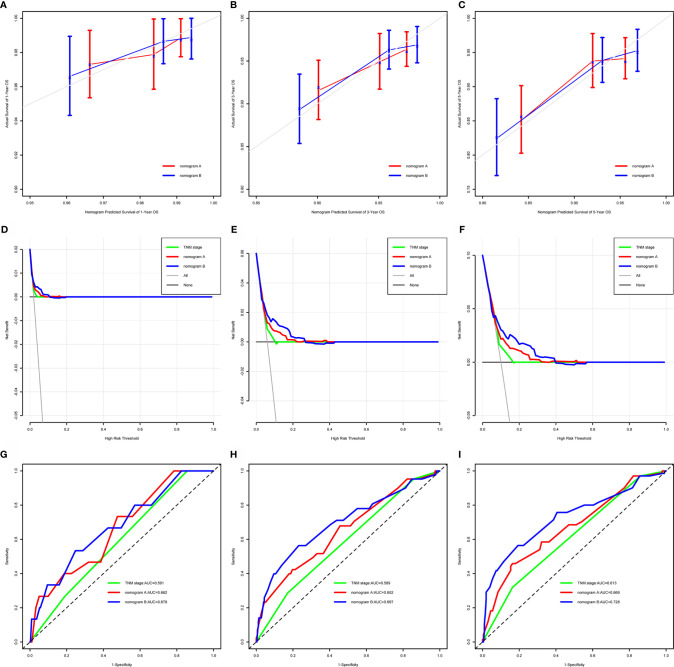
Calibration curves, decision curves, and time-dependent receiver-operating characteristic curves (tROC) to predict 1-, 3- and 5-year overall survival (OS). OS = overall survival; AUC = area under the receiver-operating characteristic curve. **(A–C)** Calibration curves for 1-, 3- and 5-year OS. Actual OS is plotted on the y-axis; nomogram-predicted probability of OS is plotted on the x-axis. The diagonal dotted line represents a perfect prediction by an ideal model. The colored solid line represents the performance of the nomogram, of which a closer fit to the diagonal dotted line represents a better prediction. **(D–F)** Decision curves for 1-, 3- and 5-year OS, the x-axis represents the threshold value, and the y-axis represents the net benefit rate after the advantages minus the disadvantages. Under the same threshold probability, a larger net benefit implies that patients can obtain the maximum benefit using the diagnosis of this model. **(G–I)** Time-dependent receiver-operating characteristic curves for 1-, 3- and 5-year OS, the x-axis represents the sensitivity value (true positive rate), and the y-axis represents the 1-specificity (false positive rate). A larger AUC implies that patients can obtain the maximum prediction using this model.

Nomogram A was constructed using histology, EBV-DNA, N stage, and T stage to predict 1-, 3-, 5- year OS (considering that P = 0.062 is very close to significance) in model 1 without S-E grade (**Figure 3**). In **Figures 4A–C**, the y-axes represent the observed survival as assessed using Kaplan–Meier analysis, the x-axes represent the predicted survival derived using the nomogram, and the solid lines are the ideal reference line along which actual survival corresponds with predicted survival. Using the calibration plot, we demonstrated that the probability of 1-, 3-, 5- year OS post-treatment indicated optimal agreement between the predicted and actual values from nomogram A. Model 2 comprised new nomogram B (with S-E grade) combined with the risk factors used in model 1 (**Figure 3**). Similarly, optimal agreement was demonstrated between the actual and predicted values for nomogram B using the calibration plots (**Figures 4A–C**).

### Comparing the Accuracy of Prediction Between Nomogram A and Nomogram B

We compared the power of prediction for survival among nomogram A, nomogram B, and conventional stage systems. The C-index of nomogram A was 0.676 (95% CI, 0.603–0.750), which was higher than that of the current staging system (0.604; 95% CI, 0.544–0.664, P = 0.006). The C-index of nomogram B was 0.717 (95% CI, 0.643–0.791), which was higher than those of nomogram A and the current staging system (0.676 (95% CI, 0.603–0.750, P = 0.020) and 0.604 (95% CI, 0.544–0.664, P < 0.001), respectively) (**Supplementary Table S1**). As shown in **Figures 4C, D**, the results of DCA and tROC demonstrated that the application of the nomogram B provided a better prediction effect than nomogram A. Thus, the nomograms with or without S-E grade showed better accuracy to predict survival than the current staging system, and suggested that the nomogram developed with the S-E grade was more useful in clinical decision-making.

### Performance of the Nomogram in Patient Risk Stratification

**Figure 5:**
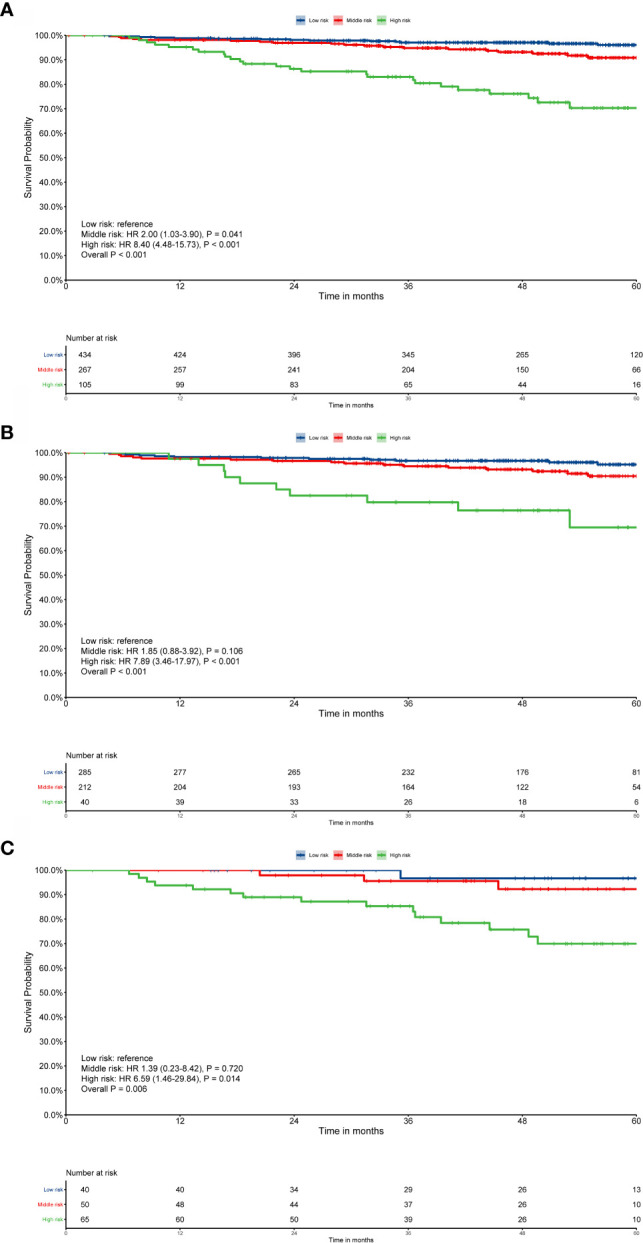
Risk group stratification within each TNM stage. **(A)** All patients; **(B)** stage III; **(C)** stage IV. Survival curves were calculated using the Kaplan–Meier method and compared using the log-rank test.

The risk score for each patient was calculated using nomogram B and the patients were sorted by total score (score: < 13.21, low risk; 13.21 to 16.42, middle risk; and >16.42, high risk). After applying the cutoff values, the patents could be stratified into different risk subgroups, which permitted survival outcomes within different TNM stages to be distinguished using Kaplan–Meier curves (all stages, P < 0.001; stage III, P < 0.001; stage IV, P = 0.002, **Figure 5**). For stage II patients (n = 204), 109 patients were in the low risk group, while only five patients were in the middle risk group. Considering the small number of patients in the middle risk group, survival analysis was not performed.

## Discussion

We developed a new model, based on our constructed parameter, called the S-E grade, which combines sarcopenia and EBV-DNA levels, to predict OS in patients with NPC. The predictive power of S-E grade was compared with that of the traditional TNM staging system. The results showed that the S-E grade predicted OS more accurately than TNM staging. The new developed nomogram B, which incorporated the S-E grade, N stage, T stage, and histology, demonstrated increased predictive accuracy compared with that of the traditional nomogram A, which included EBV-DNA, histology, N stage, and T stage, and the current TNM staging system. The conventional staging system is associated with several controversies: The current staging system only takes into account the anatomical extent of the disease, and does not comprehensively account for the biological heterogeneity of patients with NPC, nor does its consider other risk factors. Thus, for patients with NPC, such issues might affect the accuracy of prediction of conventional systems. Interestingly, our results showed that the C-indices and AUC of our constructed S-E grade were higher compared with those of the current staging system, and the C-indices of both nomogram B and nomogram A were correspondingly higher than those of current staging system; thus, the method addressed the concerns mentioned above.

In recent years, sarcopenia has been demonstrated as a good marker for prognosis of various cancers (25, 26). Many studies have shown that for patients with HNC receiving definitive (chemo)radiotherapy, sarcopenia is an independent prognostic factor for worse survival outcome (13). Huang et al. found that patients often experience different levels of muscle loss during CCRT, and severe skeletal muscle loss could shorten OS in NPC (12). Moreover, some researchers found that the presence of sarcopenia was not associated with survival (27), possibly because they determined sarcopenia according to the third lumbar vertebral level, which not be as accurate as that determined using the C3 level. Our previous article found that sarcopenia was a promising indicator for predicting clinical outcomes in patients with NPC receiving CCRT (28). Currently, there are no studies that include sarcopenia into the prognosis system of NPC, so we sought to determine whether the prediction accuracy could be improved if we included sarcopenia as a factor. Therefore, in this study, we mainly explored the feasibility of including sarcopenia into the NPC prognosis model, and compared it with the accepted model, finding that adding sarcopenia can indeed improve the prediction effect. Also, plasma EBV-DNA levels are a good biomarker in the clinical management of NPC, and since the beginning of the 21^st^ century, the EBV-DNA level has been considered the most useful biomarker for patients with NPC (29). Many studies have demonstrated that using cutoff values of 1,500 copies/ml and 4,000 copies/ml, EBV-DNA levels allow good prognostic stratification (30–32). However, sarcopenia and EBV-DNA content cannot be effectively incorporated into the TNM staging system. Interestingly, in the present study, we constructed novel S-E grade, and developed the corresponding nomogram, which incorporated sarcopenia and the EBV-DNA level into the TNM staging system, resulting in superior predictive accuracy of the nomogram compared with that of the conventional TNM staging system, thus making the EBV-DNA level and sarcopenia more clinically applicable.

Most studies have used routine clinical diagnostic CT scans to determine the skeletal muscle mass of the third lumbar vertebral segment. Unfortunately, abdominal CT scans, including third lumbar vertebral, are not routinely used for NPC. Therefore, the lack of widely available diagnostic tools to determine sarcopenia might lead to a lack of adequate research on NPC. Therefore, the majority of centers do not apply sarcopenia routinely and measurement methods are not standardized globally. The traditional nomogram A (without sarcopenia) was developed according to the classic research of Tang et al. (20). Compared with the current staging system, our developed nomogram also showed greater accuracy of prediction. Our data suggested that nomogram A remains useful for centers that do not assess sarcopenia in clinical practice. However, our newly developed nomogram B (with sarcopenia) showed the best predictive accuracy compared with traditional nomogram A and the current staging system. Our data showed that the repeatability and reliability of the established nomogram were supported by the good agreement between the predicted and actual observations in the calibration plots. Adding sarcopenia to nomogram B produced an added value of 0.041 (P = 0.020) over nomogram A. More importantly, the stratification of patients into three risk groups in the same TNM stage using the total score from nomogram B, allowed patients to be separated into groups with distinct survival outcomes. However, for patients with stage II diease, the differentiation was poor. This might have been caused by the relatively early stage of patients with stage II diease, and that the relatively high intensity of CCRT treatment results in a very good prognosis (33). In addition, for patients with stage II disease (n = 204), 109 patients were in the low risk group, while only 5 patients were in the middle risk group. This small number of cases in the middle risk group might lead to inaccurate data analysis; therefore, we are planning to further explore this in a larger sample size of patients with stage II disease.

As far as we know, this nomogram is the first to incorporate sarcopenia and EBV-DNA content to predict the survival of patients with NPC receiving CCRT. Both clinicians and patients could achieve a prediction of individual survival post-treatment *via* this simple scoring system. Choice of treatment and care options might be improved if subgroups of patients with different risks of poor survival could be identified. There is controversy surrounding the selection of patients who need intensive follow-up or additional therapy, and the developed scoring system might aid clinicians to resolve such issues. This nomogram could also provide data for patient stratification when designing clinical studies, allowing more balanced study arms to be designed. The developed nomogram is believed to be a more precise prognostic model than TNM staging and other previously used prognostic models.

There are some limitations in our study. First, we established the nomogram using data obtained only from one center based in an endemic area, thus the determination of sarcopenia still needs to be globally standardized and further external validation is needed. Second, this study only assessed non-metastatic NPC in patients treated with CCRT; as such, our conclusions were based on the particular characteristics of these patients. Third, more research is required to validate the cutoff value established in this study.

## Conclusion

The present study constructed and validated a nomogram to predict the survival of patients with NPC patients receiving CCRT. Using this model, clinicians will be able to estimate more precisely the survival of individual patients post-CCRT and identify patient subgroups that require a specific treatment strategy.

## Data Availability Statement

The authenticity of this article has been validated by uploading the key raw data on to the Research Data Deposit public platform (www.researchdata.org.cn), with the approval RDD number, RDDA2020001682.

## Ethics Statement

All procedures performed in studies involving human participants were in accordance with the ethical standards of the institutional and/or national research committee and with the 1964 Helsinki declaration and its later amendments or comparable ethical standards. This study was approved by the Clinical Research Ethics Committee of SYSUCC (number: GZR2017-224). Informed consent was obtained from all individual participants included in the study.

## Author Contributions

Conceptualization: XHua, H-XL, Z-YY, and LG. Data curation: XHua, W-ZL, XHuang, and WW. Formal analysis, XHua and W-ZL. Funding acquisition: LG and H-XL. Investigation: XHua, XHuang, H-YH, WW, and Z-QL. Methodology: XHua and W-ZL. Project administration: LG, Z-YY, and H-XL. Software: XHua, W-ZL, XHuang, and WW. Supervision: LG, Z-YY, and H-XL. Validation: XHua, XHuang, H-YH, and WW. Visualization: XHua, W-ZL, and XHuang. Writing (original draft): XHua. Writing (review and editing): all authors. All authors contributed to the article and approved the submitted version.

## Funding

This work was partly supported by the National Natural Science Foundation of China (nos. 81772877, 81773103, and 81572848).

## Conflict of Interest

The authors declare that the research was conducted in the absence of any commercial or financial relationships that could be construed as a potential conflict of interest.
