# Supplementary material for: Modeling Sarcopenia to Predict Survival for Patients With Nasopharyngeal Carcinoma Receiving Concurrent Chemoradiotherapy
Source: Front Oncol. 2021 Mar 11;11:625534. doi: 10.3389/fonc.2021.625534 (PMC7993198; doi:10.3389/fonc.2021.625534)
Supplement: Supplementary file 1 [file DataSheet_1.docx]

**Supplementary Materials**

**Supplementary Methods**

**Inclusion criteria and exclusion criteria**

The following inclusion criteria were used: (1) histological and radiographic findings confirmed the presence of non-metastatic NPC; (2) age ≥18 years; ECOG of 0 to 2; (3) the patients underwent radical intensity-modulated radiotherapy plus weekly or triweekly cisplatin concurrent chemotherapy; and (4) adequate hematologic, renal, and hepatic function. The following exclusion criteria were used: (1) history of previous anticancer therapy (n=4); (2) pregnancy and lactation (n=3); (3) a history of previous or synchronous malignant tumors (n=5); (4) The presence of a primary distant metastasis (n=2); (5) age <18 years (n=6); (6) inadequate hematologic, renal, and hepatic function (n=8) (7) lack of follow-up or incomplete clinical data (n=211).

**The methodology of muscle contours**

The single axial CT-slide at level of C3 first showing the entire vertebral arc when scrolling from caudal to cranial direction was selected. To avoid over- or underestimation of skeletal muscle area, the Hounsfield unit (HU) settings ranged from -29 to +150 HU. Outer contours of both sternocleidomastoid and paravertebral muscles were delineated manually (Figure S1), and the skeletal muscle area was computed automatically within the contoured perimeters. All CT-scans were delineated by an experienced NPC radiation oncologist (LG).

**The methodology of detecting plasma EBV DNA**

Samples of peripheral blood (3 ml) were collected in an EDTA tube from all NPC patients and were centrifuged at 1600 x g for 15 min for isolation of plasma and PBC. Plasma DNA was extracted using the QIAamp Blood Kit (Qiagen, Hilden, Germany) and was stored at -80°C until further processing. A total of 500 μl of the plasma samples were used for DNA extraction per column and a final elution volume of 50 μl was used to elute the DNA from the extraction column. A real-time quantitative PCR system was developed for plasma EBV DNA detection toward the BamHI-W region of the EBV genome. The sequences consisted of the amplification primers W-44F (5’-AGT CTC TGC CTC AGG GCA-3’) and W-119R (5’-ACA GAG GGC CTG TCC ACCG-3’) and the dual-labeled fluorescent probe W-67T (5’-[FAM] CAC TGT CTG TAA AGT CCA GCC TCC[TAMRA]-3’).

**Treatment for NPC patients**

CCRT protocol comprised a triweekly (80-100 mg/m^2^) or weekly (30-35mg/m^2^) administration of cisplatin concurrently with intensity-modulated radiation therapy (IMRT). If only two cycles of concurrent chemotherapy were completed during the radiotherapy phase, then the third cycle of concurrent chemotherapy was given within 1 week after completion of radiotherapy, If the third cycle of chemotherapy was not administered in this time, it was not given. In this study, 60% (483) patients received two cycles concurrent chemotherapy, 40% (323) patients received three cycles concurrent chemotherapy. All patients at the study institution were treated according to the principle of treatment for NPC patients at SYSUCC. Radiotherapy quality assurance (QA) was performed before radiotherapy for all the NPC patients. Details of the radiotherapy techniques used at the SYSUCC were described in previous studies. (1. Zhao C, Han F, Lu LX, et al. Intensity modulated radiotherapy for local-regional advanced nasopharyngeal carcinoma. Ai Zheng 2004;23(11 Suppl):1532-7; 2. Ma J, Liu L, Tang L, et al. Retropharyngeal lymph node metastasis in nasopharyngeal carcinoma: prognostic value and staging categories. Clin Cancer Res 2007;13(5):1445-52.)

**Figure S1.** Slice of computed tomography (CT) simulation images of skeletal muscle at the level of the third cervical vertebra (C3).

We measured the cross-sectional area of the sternocleidomastoid and paravertebral muscles on an axial slice at the level of C3 vertebrae; the CT Hounsfield unit thresholds were −29 to +150 for skeletal muscle.


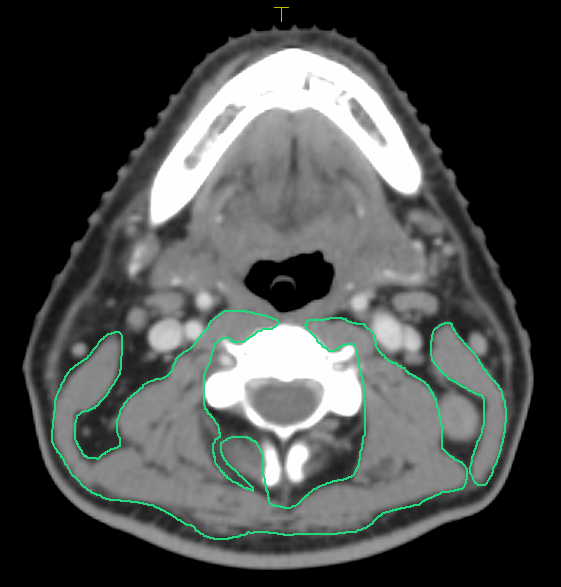


**Figure. S2.** Kaplan­–Meier survival curves for RFS and DMFS.

Kaplan–­Meier curves for: **(A)** RFS; **(B)** DMFS.

**Figure. S2A**


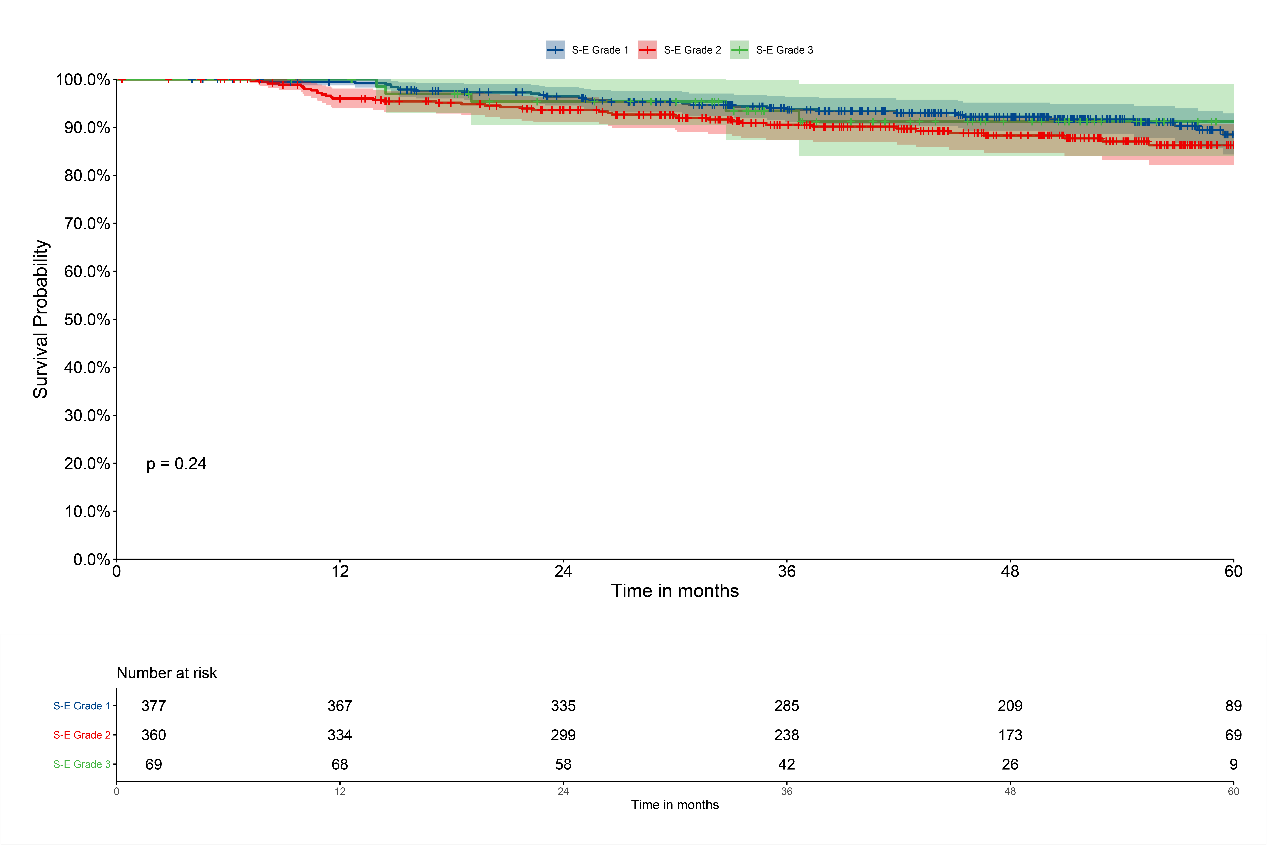


**Figure. S2B**


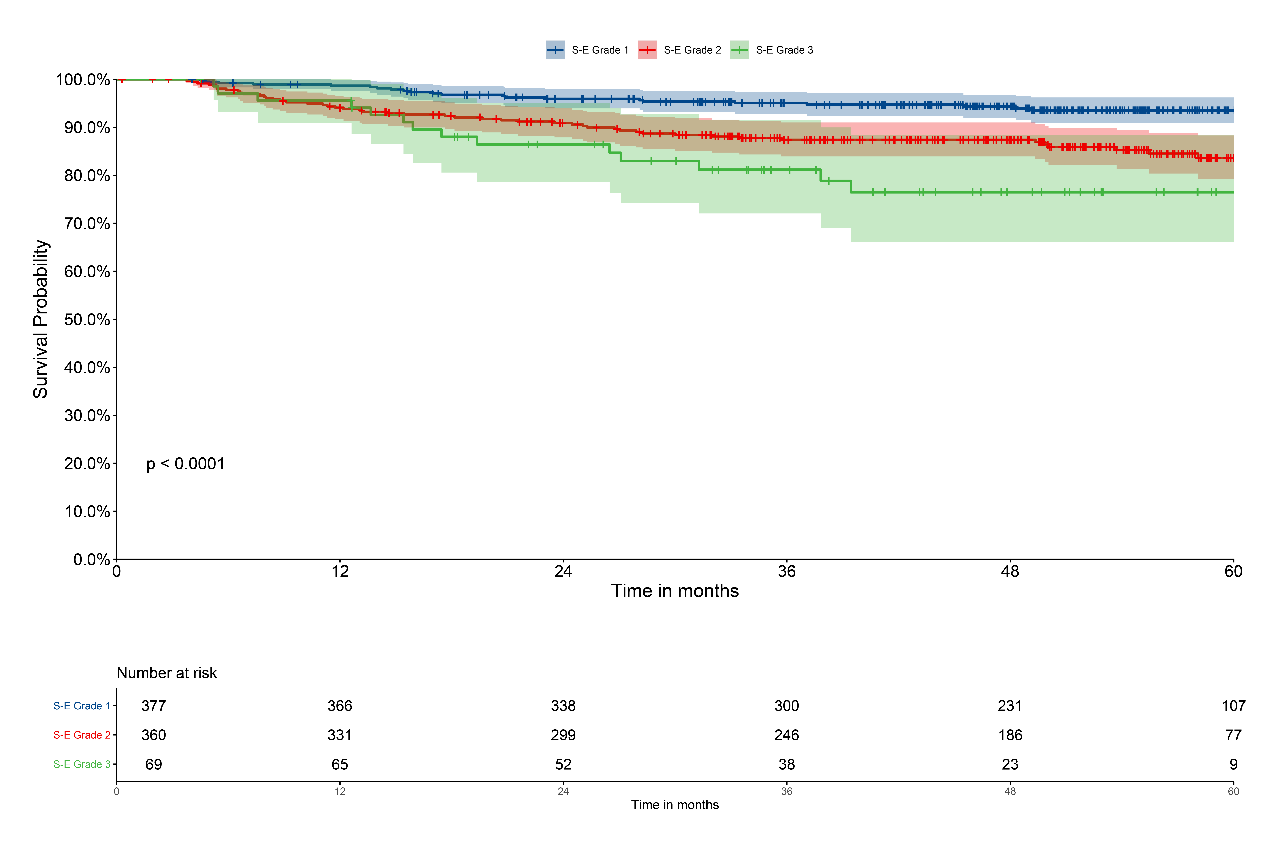


Survival curves were calculated using the Kaplan–Meier method and compared using the log-rank test.

**Table S1**. The C-index of S-E grade, TNM stage, sarcopenia, EBV-DNA, nomogram A and nomogram B to predict overall survival.

| **Characteristic** | **C-index (95%CI)** | **P** |
| --- | --- | --- |
| S-E grade | 0.663 (0.594–0.732) |  |
| TNM stage | 0.604 (0.544–0.664) |  |
| Sarcopenia | 0.623 (0.558–0.688) |  |
| EBV-DNA | 0.600 (0.535–0.665) |  |
| nomogram A | 0.676 (0.603–0.750) |  |
| nomogram B | 0.717 (0.643º0.791) |  |
| S-E grade vs TNM stage |  | 0.010 |
| S-E grade vs sarcopenia |  | 0.134 |
| S-E grade vs EBV-DNA |  | 0.012 |
| nomogram B vs nomogram A |  | 0.020 |
| nomogram B vs TNM stage |  | ＜0.001 |
| nomogram A vs TNM stage |  | 0.006 |

P values are calculated based on normal approximation using function rcorrp.cens in Hmisc package.

Abbreviations: C-index = concordance index; CI = confidence interval; S-E grade = sarcopenia EBV-DNA grade; EBV-DNA = Epstein-Barr virus DNA.
